# Stably bridging a great divide: localization of the SpoIIQ landmark protein in *Bacillus subtilis*

**DOI:** 10.1111/mmi.12365

**Published:** 2013-08-23

**Authors:** Lotte Søgaard-Andersen

**Affiliations:** Max Planck Institute for Terrestrial MicrobiologyKarl-von-Frisch Str. 10, 35043, Marburg, Germany

## Abstract

Many bacterial proteins involved in fundamental processes such as cell shape maintenance, cell cycle regulation, differentiation, division and motility localize dynamically to specific subcellular regions. However, the mechanisms underlying dynamic protein localization are incompletely understood. Using the SpoIIQ protein in *Bacillus subtilis* as a case study, two reports present important novel insights into how a protein finds its right place at the right time and remains stably bound. During sporulation, SpoIIQ localizes in clusters in the forespore membrane at the interface that separates the forespore and mother cell and functions as a landmark protein for SpoIIIAH in the mother cell membrane. The extracellular domains of SpoIIQ and SpoIIIAH interact directly effectively bridging the gap between the two membranes. Here, SpoIIQ localization is shown to depend on two pathways, one involves SpoIIIAH, the second involves two peptidoglycan-degrading enzymes SpoIIP and SpoIID; and, SpoIIQ is only delocalized in the absence of all three proteins. Importantly, in the absence of SpoIIIAH, SpoIIQ apparently localizes normally. However, FRAP experiments demonstrated that SpoIIQ is not stably maintained in the clusters in this mutant. Thus, a second targeting pathway can mask significant changes in the localization of a protein.

## Introduction

Due to a large extent to technological breakthroughs in high-resolution microscopy, bacterial cells have become amenable to the investigation of cell biological questions. These investigations have revealed that higher-order spatial organization, once thought to be a property specific to eukaryotic cells, are also important characteristics of bacterial cells (Gitai *et al*., 2005; Shapiro *et al*., 2009). This organization can be highly dynamic over time, and an astonishing array of different spatiotemporal protein localization patterns has been uncovered ranging from proteins with a relatively stationary localization, proteins with dynamic localization and changing localization in a cell cycle-dependent or cell cycle-independent manner, proteins forming gradients, to proteins that oscillate rapidly over the chromosome or between the cell poles. How these patterns are established and maintained are areas of intense investigations. Two reports from the groups of Kit Pogliano (Fredlund *et al*., 2013) and David Rudner (Rodrigues *et al*., 2013) present important novel insights into how the SpoIIQ landmark protein in *Bacillus subtilis* finds its right place at the right time during sporulation.

Distinct spatiotemporal localization patterns have been observed for different types of macromolecules in bacteria, including the chromosome (Wang *et al*., 2013), mRNAs (Nevo-Dinur *et al*., 2012), proteins involved in processes such as cell shape maintenance, cell cycle regulation, differentiation, division and its regulation (Shapiro *et al*., 2009), motility and its regulation (Bulyha *et al*., 2011) as well as metabolic multi-enzyme complexes (Straight *et al*., 2007), lipids (López and Kolter, 2010) and a second messenger (Christen *et al*., 2010), and even protein- and lipid-bounded organelles exist in bacteria (Murat *et al*., 2010). Thus, establishing and maintaining spatial organization with the asymmetric localization of macromolecules is a fundamental issue in bacteria.

Major questions in understanding the spatiotemporal dynamics of bacterial cells are how proteins find their correct localization and how this localization may change over time. Relatively little is known about how bacterial proteins become localized. A mechanism involving the recognition of a geometric cue was proposed for the peripheral membrane proteins SpoVM (Ramamurthi *et al*., 2009) and DivIVA (Lenarcic *et al*., 2009; Ramamurthi and Losick, 2009) in *B. subtilis*. In this model, SpoVM and DivIVA interact specifically with membranes of positive and negative curvature respectively. Alternatively, the localization of a protein X may depend on a different protein that acts as a landmark for X (Shapiro *et al*., 2009) as has been shown for several membrane proteins that diffuse through the membrane until captured by a localized landmark protein in a diffusion-and-capture mechanism (Rudner *et al*., 2002; Deich *et al*., 2004) as well as for several cytoplasmic proteins (Bowman *et al*., 2008; Ebersbach *et al*., 2008; Yamaichi *et al*., 2012). Also, some localized proteins, e.g. MipZ in *Caulobacter crescentus* (Thanbichler and Shapiro, 2006), interact with proteins that bind to specific sites on the chromosome. Given that the *C. crescentus* chromosome is spatially highly organized (Viollier *et al*., 2004) this interaction results in localization of the interacting protein. Finally, several ParA/MinD proteins have been shown to self-organize in a nucleotide-dependent manner on a scaffold, i.e. the cytoplasmic membrane or the chromosome, in that way positioning other proteins correctly (Lutkenhaus, 2012).

## Sporulation in *B. subtilis*

**Fig. 1:**
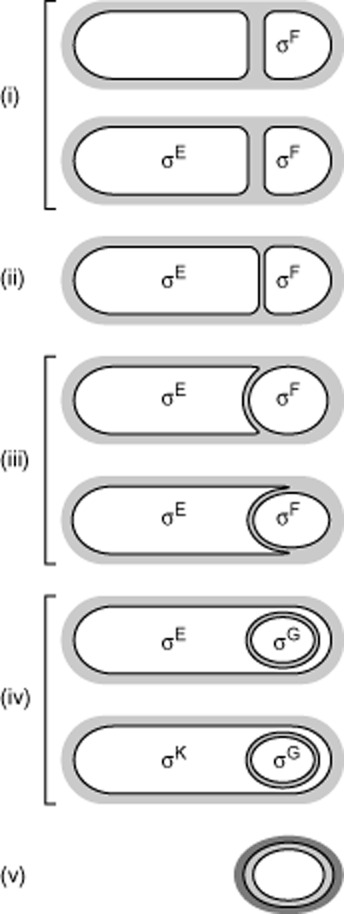
Morphological changes and compartment specific σ-factors during *B. subtilis* endospore formation. (i) The polar septum forms with a thick layer of PG separating the mother cell and the forespore. PG is indicated in light grey. σ^F^ becomes active in the forespore and initiates a signalling pathway that leads to activation of σ^E^ in the mother cell. (ii) The septal PG is thinned. (iii) During engulfment, the forespore is released from the cell wall by PG hydrolysis and the mother cell membrane migrates in the wake of the hydrolysed PG. (iv) σ^G^ becomes active in the developing spore after completion of engulfment and initiates a signalling pathway that leads to activation of σ^K^ in the mother cell. (v) Cortex forms between the inner and outer spore membranes and coat proteins (dark grey) assemble on the spore surface. The mother cell lyses and the mature endospore is released.

Sporulation in *B. subtilis* has served as an important model system to understand how proteins find their correct localization at the correct time because a variety of the proteins important for the sporulation process including morphogenesis of the endospore as well as gene expression localize dynamically to the interface between the mother cell and the forespore. *B. subtilis* initiates sporulation in response to nutrient limitation and the sporulation process proceeds in well-defined morphological stages (Kroos, 2007) (Fig. 1). A key event in sporulation is the formation of an asymmetrically localized septum consisting of a thick layer of peptidoglycan (PG) that divides the developing cell into a small forespore and a larger mother cell (Fig. 1i). Initially, the two cells lie side-by-side separated by a thick layer of PG (Fig. 1i). But later, and after the septal PG has thinned (Fig. 1ii), the mother cell membrane starts to migrate around the forespore in a process referred to as engulfment (Fig. 1iii), and in which the forespore is essentially ‘cut-free’ of the cell wall PG. Eventually, the spore is completely engulfed and pinched off as a free protoplast that is enclosed within the mother cell (Fig. 1iv). The protoplast contains two membranes: The cytoplasmic membrane is derived from the forespore and the outer membrane is derived from the mother cell and separated from the cytoplasmic membrane by a thin layer of PG (Tocheva *et al*., 2013). After formation of the free protoplast, a thick cortex of new PG is produced in the space between these two membranes and coat proteins are deposited on the surface of the spore. Ultimately, the mother cell lyses and the mature endospore is released (Fig. 1v).

Throughout this developmental programme the forespore and the mother cell follow distinct but highly co-ordinated programmes of gene expression that depend on the sequential activation of compartment-specific σ-factors (Rudner and Losick, 2001; Kroos, 2007) (Fig. 1). In the forespore, σ^F^ becomes active after formation of the asymmetric septum. σ^F^ activity, in turn, leads to activation of σ^E^ in the mother cell in a process that depends on an intercellular communication pathway from the forespore to the mother cell. In a compartment-specific manner, σ^F^ and σ^E^ direct the synthesis of distinct sets of proteins required for engulfment of the forespore as well as two σ-factors, σ^G^ in the forespore and σ^K^ in the mother cell. After completion of the engulfment process, σ^G^ is activated in the prespore. σ^G^ activity, in turn, leads to activation of σ^K^ in the mother cell in a process that depends on an intercellular communication pathway from the prespore to the mother cell.

## The engulfment process

The two papers from the Pogliano and Rudner groups focus on how proteins involved in the engulfment process and σ^G^ activation find their correct subcellular localization. The engulfment process depends on two sets of proteins most of which are exclusively synthesized in the mother cell and one that is exclusively synthesized in the forespore. Importantly, these proteins have distinct subcellular localizations that change dynamically as engulfment proceeds. Initially, they localize to the asymmetric septum and later, as engulfment proceeds, some only localize to the advancing leading edges of the engulfing membrane at the mother cell/forespore interface and some in addition also localize in foci at the mother cell/forespore interface behind the leading edges of the engulfing membrane.

**Fig. 2:**
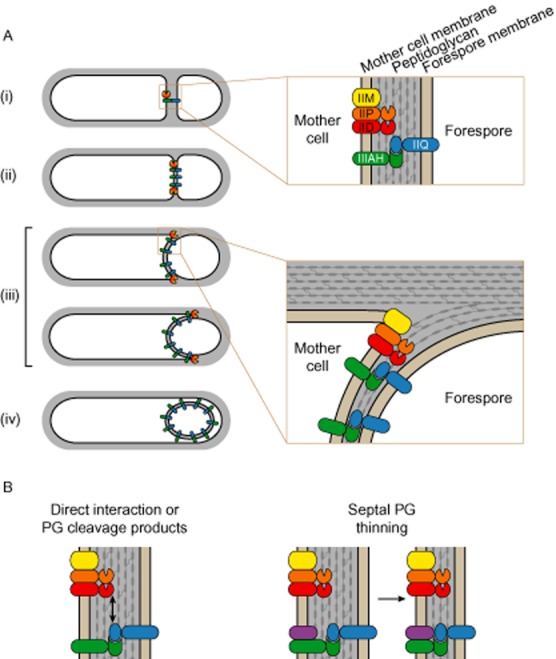
Localization of IIQ and IIIAH as well as IIM, IIP and IID during engulfment. A. (i) Initially IIQ and IIIAH as well as IIM, IIP and IID localize to the midpoint of the septum with the thick layer of PG. For simplicity, the remaining seven SpoIIIA are not shown. Box indicates region expanded on the right. Membranes are indicated in light brown and PG in light grey. (ii) During septal PG thinning, IIM, IIP and IID relocate to the leading edges of the engulfing mother cell membrane and IIQ and IIIAH colocalize at the mother cell/forespore interface at the leading edges of the engulfing mother cell membrane as well as in foci along the remaining mother cell/forespore interface. (iii) During engulfment, IIM, IIP and IID as well as IIQ and IIIAH localize to the leading edges of the engulfing mother cell membrane and additional IIQ•AHIII foci are formed along the expanding mother cell/forespore interface. Box indicates region expanded on the right. Colour code is as in (i). (iv) After completion of engulfment, the IIQ•IIIAH complex together with the remaining seven SpoIIIA proteins form a secretion system that spans from the mother cell to the developing spore and ‘nurtures’ the spore. B. Models for how IIM, IIP and IID assist in stable localization of IIQ. Left panel: IIM, IIP and IID function to localize IIQ by one or more of the three proteins directly interacting with IIQ or IIQ interacts with their PG cleavage products. Right panel: IIM, IIP and IID function indirectly in IIQ localization by degrading PG. PG degradation allows the extracellular LytM domain of IIQ to interact with an unknown mother cell encoded protein (marked in purple).

One set of proteins consists of the integral membrane proteins SpoIIM, SpoIIP and SpoIID (henceforth, IIM, IIP and IID). These three proteins are essential for engulfment and σ^G^ activation, function to thin the septal PG and hydrolyse PG during the engulfment process, are synthesized in the mother cell, and interact to form a complex that localizes to the mother cell derived outer forespore membrane (Fig. 2A). IIM has no known enzymatic activity whereas IIP and IID degrade PG (Abanes-De Mello *et al*., 2002; Chastanet and Losick, 2007). Specifically, IIP has d, d-endopeptidase and amidase activity and IID is a lytic transglycosylase that only acts on peptide-free, ‘denuded’ glycan strands (Morlot *et al*., 2010). IIM is recruited by the SpoIIB landmark protein to the midpoint of the septum. Here, it recruits IIP, which, in turn, recruits IID (Abanes-De Mello *et al*., 2002; Aung *et al*., 2007; Chastanet and Losick, 2007). IIP and IID have been suggested to act sequentially with IIP removing peptide cross-links and peptides while IID cleaves the ‘denuded’ glycan strands (Morlot *et al*., 2010). In parallel with thinning of the septal PG by IIM, IIP and IID, the three proteins spread throughout the septum and become localized at the leading edges of the engulfing mother cell membrane (Abanes-De Mello *et al*., 2002) (Fig. 2Aii–iii). These findings together with the observation that IIM, IIP and IID are required for the movement of the engulfing membrane around the forespore have led to a model in which these three proteins function as a motor to drag the mother cell membrane around the forespore in a process in which the directed PG hydrolysis provides directionality to the migrating mother cell membrane (Abanes-De Mello *et al*., 2002).

The second set of proteins with an important function in engulfment and σ^G^ activation consists of the forespore encoded SpoIIQ (henceforth, IIQ) and the eight mother cell encoded proteins of the *spoIIIA* locus (Fig. 2A). IIQ is an integral membrane protein that initially localizes to the septum in the forespore membrane (Rubio and Pogliano, 2004). As the engulfment process proceeds, IIQ in the forespore membrane tracks with the leading edges of the engulfing mother cell membrane and also localizes in foci at the mother cell/forespore interface behind these leading edges (Rubio and Pogliano, 2004) (Fig. 2Aii–iii). IIQ localization involves the directed insertion in the septum and capture by a mother cell encoded protein (see details below) (Rubio and Pogliano, 2004).

Among the eight proteins encoded by the *spoIIIA* locus, several show homology to proteins of type III and IV secretion systems. SpoIIIAH (henceforth, IIIAH) localizes to the septum by a diffusion-and-capture mechanism in which a direct interaction between its extracellular domain and the extracellular domain of IIQ captures IIIAH and these two proteins colocalize throughout the engulfment process (Blaylock *et al*., 2004; Doan *et al*., 2005) (Fig. 2Ai–iv). Thus, a forespore encoded protein recruits a mother cell protein by an interaction across the septal space. The IIQ•IIIAH complex is only essential for engulfment under certain conditions, i.e. in mutants with reduced IIM, IIP and IID activity or when the PG has been artificially removed (Broder and Pogliano, 2006). In total, these findings have led to the suggestion that two pathways confers robustness to the engulfment process, one involves the IIM•IIP•IID complex and one involves the IIQ•IIIAH complex (Broder and Pogliano, 2006).

In addition to being involved in engulfment, the IIQ•IIIAH complex recruits the remaining seven SpoIIIA proteins to form a multiprotein complex that bridges the two membranes surrounding the forespore and the gap between them and is essential for σ^G^ activation following completion of engulfment (Doan *et al*., 2005; 2009). Thus, whereas IIQ•IIIAH is dispensable for engulfment, this complex is absolutely essential for σ^G^ activation and in this way for sporulation. This multiprotein complex has been suggested to serve as a secretion system or ‘feeding tube’ between the mother cell and the developing spore and through which the mother cell nurtures the protoplast to maintain its metabolic potential after it becomes sequestrated within the mother cell (Camp and Losick, 2008; Meisner *et al*., 2008; Camp & Losick, 2009; Doan *et al*., 2009; Levdikov *et al*., 2012).

## SpoIIQ localization, the new findings

Previous analyses showed that IIQ localizes to the septum in a manner that depends on σ^E^ activity in the mother cell (Rubio and Pogliano, 2004). Curiously, however, this localization was reported to be independent of its interaction partner IIIAH in the mother cell (Blaylock *et al*., 2004). Therefore, a major question has been how IIQ becomes localized. An important part of the answer to this question has now been provided in the two papers from the Pogliano and Rudner groups.

Using a combination of genetics and cell biology approaches, Fredlund *et al*. (2013) and Rodrigues *et al*. (2013) report that IIQ localization depends on two separate pathways, one involves the direct interaction between IIQ and IIIAH and the second depends on the engulfment proteins IIM, IIP and IID. In the absence of IIIAH, IIQ still formed foci resembling those in the wild type. In the absence of IIM, IIP and IID, IIQ also localized to the septum. However, in the absence of all four proteins (IIIAH as well as IIM, IIP and IID), IIQ localized throughout the forespore membrane and was no longer enriched at the septum. Importantly, and a lesson to learn from for all cell biologists, fluorescence recovery after photobleaching (FRAP) experiments showed that IIQ in the absence of IIIAH was no longer stably maintained in individual foci but rapidly exchanged with IIQ in neighbouring foci (Fredlund *et al*., 2013). Moreover, FRAP experiments showed that in the absence of IIM, IIP and IID, IIQ localized stably to the septum indicating that immobilization is independent of IIM, IIP and IID and depends on the interaction with IIIAH. Similarly, in FRAP experiments IIIAH was found to localize stably in foci in a manner that depended on IIQ but not on IIM, IIP and IID. Thus, once an IIQ•IIIAH complex has formed spanning the two membranes surrounding the forespore, the two proteins are stably maintained in this complex with little or no exchange with neighbouring complexes.

How, then, do IIM, IIP and IID stimulate IIQ localization? To distinguish whether IIQ interacts directly with IIM, IIP and/or IID, with their PG degradation products, or an unknown protein (which would not be able to interact with IIQ in the absence of IIM, IIP and IID due to the thick PG layer in the septum) Fredlund *et al*. analysed the effect of a catalytic IID mutant that is unable to cleave PG and fails to support septal thinning and engulfment. Based on the analysis of this mutant, they suggest that IIQ interacts with one or more of the IIM, IIP and IID proteins or with their PG cleavage products (Fig. 2B, left). Rodrigues *et al*. analysed the effect of catalytic IIP and IID mutants on IIQ localization and observed that catalytic activity of IIP as well as of IID is important for IIQ localization supporting a scenario in which localization of IIQ requires the PG degrading activities of IID and IIP rather than utilizing protein–protein interaction across the septum. Furthermore, induction of IID, IIP and IIM synthesis in cells lacking σ^E^ failed to localize IIQ. Interestingly, the extracellular domain of IIQ contains a degenerate LytM domain (Camp and Losick, 2009; Levdikov *et al*., 2012; Meisner *et al*., 2012). Because this degenerate LytM domain appears to be unable to bind PG (Rodrigues *et al*., 2013), Rodrigues *et al*. suggest that IIP and IID promote IIQ localization by degrading PG and that this degradation would allow the extracellular LytM domain of IIQ to interact with an unknown mother cell encoded protein (Fig. 2B, right). Moreover, they identify residues in the LytM domain of IIQ that could provide an interaction interface with this unknown protein. Thus, the exact mechanism by which IIM, IIP and IID promotes the stable interaction with IIIAH remains to be discovered.

## Lessons learned

Two lessons can be learned from these two reports. First, even though a protein apparently localizes normally in the absence of an interaction partner, the dynamics and turnover of the protein can be dramatically altered – an effect that is not uncovered by traditional epifluorescence microscopy but depends on using *in vivo* imagining technologies that allow protein dynamics to be followed with high temporal resolution. Second, these new results teach us that the mechanism(s) underlying the localization of a particular protein might rely on more than just a single targeting factor. Whether other bacterial proteins also depend on several targeting factor remains to be seen.
